# Understanding External Peak Demands in Elite vs. Non-Elite Male Basketball Players

**DOI:** 10.3390/sports13060179

**Published:** 2025-06-09

**Authors:** Yoel Antoranz, Enrique Alonso-Pérez-Chao, Carlos Mª Tejero-González, Hugo Salazar, Juan del Campo-Vecino, Sergio L. Jiménez-Sáiz

**Affiliations:** 1 Department of Physical Education, Sport and Human Movement, Universidad Autónoma de Madrid, 28049 Madrid, Spain; yoel.antoranz@estudiante.uam.es (Y.A.); carlos.tejero@uam.es (C.M.T.-G.); juan.delcampo@uam.es (J.d.C.-V.); 2 Department of Physical Activity and Sports Science, University Alfonso X el Sabio, 28691 Villanueva de la Cañada, Spain; ealonsoperezchao@gmail.com; 3 Department of Real Madrid Graduate School, Faculty of Medicine, Health and Sports, Universidad Europea de Madrid, 28670 Madrid, Spain; 4 Department of Physical Activity and Sports Sciences, Faculty of Health Sciences, Euneiz University, 01013 Vitoria-Gasteiz, Spain; hsalazar002@gmail.com; 5 Faculty of Education and Sport, University of Basque Country (UPV/EHU), 48940 Vitoria-Gasteiz, Spain; 6 Sport Sciences Research Centre, Faculty of Education & Sport Sciences and Interdisciplinary Studies, Universidad Rey Juan Carlos, 28942 Fuenlabrada, Spain

**Keywords:** most demanding scenarios, PlayerLoad, team sport, worst-case scenarios, physical demands, accelerometry, athlete health management

## Abstract

Background: Understanding the physical demands of basketball at different competitive levels is essential for optimizing training and performance. While elite players are often assumed to experience the highest physical loads, evidence comparing peak external demands (PDs) between elite and non-elite athletes using time-window analysis is limited. Therefore, the aim of this study was to examine how player level (Elite vs. Non-Elite) influences the external PDs experienced by male basketball players. Methods: This study examined PlayerLoad™ (PL) PDs in elite (*n* = 10) and non-elite (*n* = 11) male basketball players during the 2023–2024 season. Players were monitored using inertial measurement units (IMUs) during official and friendly matches (elite = 8 games; non-elite = 29 games). Peak PL values were computed using rolling averages across 30-s, 1-min, and 3-min time windows. Linear mixed-effects models were employed to examine differences between groups, adjusting for age and match nature. A secondary analysis was carried out including only friendly matches. Results: Non-elite players exhibited significantly higher PL PDs across all the time windows (*p* < 0.001), with effect sizes ranging from small to very large in the main analysis (ES = −0.41 to −2.11) and from very large to extremely large in the secondary analysis (ES = −2.68 to −5.06). Differences were more pronounced in longer durations. These results suggest that non-elite athletes sustain higher physical loads, possibly due to less efficient movement patterns and lower tactical regulation. Conclusions: Elite players display lower peak external loads than their non-elite counterparts, likely due to superior cognitive, tactical, and perceptual abilities that enhance movement economy. Training programs should incorporate tasks that combine physical intensity with decision-making demands to foster efficiency and potentially reduce injury risk.

## 1. Introduction

Basketball is an intermittent sport [1,2], characterized by alternating periods of high-intensity actions and low-intensity or recovery phases [2]. The measurement and monitoring of these actions have evolved significantly over time. Initially, these were conducted through observational methods such as time–motion analysis, which required significant time and was prone to human error [1,3]. Currently, devices such as Electronic Performance Tracking Systems, which typically encompass inertial movement units (IMUs) and/or local positioning systems (LPS), are primarily used in indoor sports, as they provide valid and reliable measurements for estimating external load, particularly peak demands (PDs) or the most demanding scenarios (MDS) [3,4]. External load refers to the measurement of variables such as the number of jumps, the number and intensity of accelerations and decelerations, changes of direction, player load, distance covered at different intensities, or walking distance [4,5,6].

Basketball performance can be influenced by numerous technical, tactical, physical, mental, and psychological factors, as well as contextual elements such as the quality of the opponent, competition schedule, athlete recovery, diet, or sleep quality [7,8,9,10,11,12]. Therefore, competitive demands may also be affected by these variables.

The analysis of PDs is conducted using moving time windows, which help prevent underestimation of the values reached, as can happen when using average values [13,14]. The rolling average method is preferred, as it ensures no peak load is overlooked, unlike the fixed-window method [15]. Measurement windows can be either short (15, 30, 45 s, or 1 min), which helps identify peak load values, or long (2, 3, 4, or 5 min), allowing for comparisons with average values or training tasks [4].

An intriguing avenue for future PD analysis could be to explore whether it is influenced by the type of competition or the athlete’s level. Athlete-level classification can vary depending on criteria or population distribution, with current classifications including world-class, elite (international level), highly trained (national level), trained (local level), or recreational (active) [16]. Elite athletes are those who compete at the international level, representing approximately 0.0025% of the global population [16]. Other authors argue that athlete classification does not conform to fixed categories but rather represents a continuum in which various variables must be considered [17]. For the purpose of this study, the classification system described first will be used.

Despite its relevance, few studies in the literature have examined differences in external load—particularly PDs and MDS—between elite and non-elite basketball players. From a traditional standpoint, studies using time–motion analysis typically employ average values to compare elite and non-elite athletes. The work by Scanlan et al., which compared Australian basketball players, concluded that elite athletes experience higher external load demands during a game, although load peaks or “bursts” are greater in sub-elite athletes, with longer recovery times [18]. A similar study conducted with rugby players showed that average values are similar for both elite and semi-elite athletes, although elite players exhibit higher load demands during the first half [19]. Finally, a study conducted with basketball players compared fast break actions (successful transition from defense to offense) found no significant differences in the actions or their effectiveness between elite and sub-elite players [20].

In contrast, two studies have gone beyond time–motion analysis [21,22]. The first, which employed LPS for measurement, compared basketball players in different positions (guard, forward, and center) from an elite-level team and a semi-elite team. However, they did not use measurement windows but instead relied on average values. The results showed that sub-elite athletes performed more accelerations and decelerations than elite athletes, although, for forward players, the maximum accelerations and decelerations were of greater intensity at the elite level [21].

The second study compared two elite-level teams: one competing in the Euroleague (the top-tier European competition) and the other in the EuroCup (the second-tier European competition). Using IMU devices and measurement windows of 30 s, 60 s, and 180 s, the study found that the EuroCup team (a lower elite level) reached higher peak values in accelerations, decelerations, changes of direction, and jumps, with effect sizes ranging from 0.34 to 1.15 [22].

Moreover, understanding these physical demands has crucial implications for athlete health management. Higher external peak demands have been linked to increased injury risk [23], highlighting the importance of tailoring training to match competition demands and reduce the potential for overuse injuries and long-term physical deterioration.

As noted, there is a scarcity of research examining PDs in elite versus non-elite basketball players. Therefore, the aim of this study was to examine how player level (elite vs. non-elite) influences the external PDs experienced by male basketball players across three specific time frames (30-s, 1min, and 3-min). Based on the reviewed literature [18,20,21,22], we hypothesize that elite athletes will exhibit lower PD values compared to non-elite athletes.

## 2. Methods

### 2.1. Participants

The study sample consisted of two distinct teams, each representing differing levels of athletic proficiency. A convenience sampling method was employed, recruiting participants based on accessibility from both teams. One team comprised elite players engaged in competitive matches within the EuroLeague and Spain’s First Basketball Division. The elite players (*n* = 10, mean ± standard deviation: age: 25.9 ± 3.2 years, height: 197.5 ± 8.1 cm, body mass: 91.8 ± 4.3 kg) were closely monitored throughout 8 friendly games during the 2023–2024 season. In contrast, the non-elite team consisted of players competing in the Spanish 4th Division. These non-elite players (*n* = 11, mean ± standard deviation: age: 20.72 ± 4.2 years, height: 196.2 ± 9.9 cm, body mass: 88.8 ± 14.3 kg) were monitored during 26 official and 3 friendly games in the same season, 2023–2024. All participants signed a written informed consent form prior to participation, and the study was approved by the local ethics committee (CEI-137-2955), in accordance with the Declaration of Helsinki.

For inclusion in the final analysis, game samples required players to have completed at least 5 min of device-recorded playing time in each game. In this way, playing time included all stoppages, such as free throws, fouls, and out-of-bounds, but excluded break periods between quarters, time-outs, or moments when players were substituted [24]. Consequently, five game samples were excluded for failing to meet this criterion. Furthermore, players were only considered eligible for inclusion in the study if they had participated in at least 50% of the games (elite team = 4/8 games and non-elite team = 15/29 games). Moreover, data from any game were excluded from the analysis if a player withdrew due to injury or if the device battery was depleted during play. Overall, 300 game samples (elite = 56 samples; non-elite = 244 samples) across the 21 male players (elite = 10 players; non-elite = 11 players) were included in the analyses.

### 2.2. Design

This observational comparative study analyzed and compared PlayerLoad™ (PL) between elite and non-elite male basketball players during official and friendly matches over the 2023–2024 season. Throughout the games, each player utilized a monitoring device (S7, Catapult Sports, Melbourne, Australia) discretely integrated into a tailored neoprene vest beneath standard playing attire and positioned accurately over the upper thoracic spine, between the scapulae [25]. Each device was equipped with microsensor technology, including an accelerometer (±16 g, 100 Hz), a magnetometer (±4900 µT, 100 Hz), and a gyroscope (up to 2000 deg/s, 100 Hz). To ensure consistency and minimize potential output discrepancies, each player continued to use the same device throughout the entire study period [26].

### 2.3. Physical Variables

PDs for PL were computed in absolute terms and were extracted individually for each player across various time windows (30-s, 1-min, and 3-min). PL is a variable that represents, in arbitrary units, the sum of movement occurring in each of the planes of motion during physical activity, taking into account the squared rates of change in acceleration for each of those planes [27]. These PDs were directly derived from the Catapult software (version 3.11.0). Peak values were identified using rolling averages, which offer a more accurate approach for measuring peak demands compared to fixed time interval methods [15] and have been previously applied in basketball research [4]. Subsequently, the extracted PDs were inputted into customized Microsoft Excel spreadsheets (version 16.0, Microsoft Corporation, Redmond, WA, USA) for further analysis.

### 2.4. Statistical Analysis

All data analyzed were presented as mean values, along with their standard deviation and coefficient of variation. To assess differences in PL PDs between groups, linear mixed models were employed, followed by Bonferroni post hoc analysis. A separate linear mixed model was conducted for each time window (30-s, 1-min, 3-min), incorporating player level (elite or non-elite) as a fixed factor, the athlete as a random factor, and the interaction between player level and athlete also as a random factor. Age was included as a covariate in all models to control for its effect, added both as a fixed-effects factor and as a random-effects factor in its interaction with player level and the athlete. To account for the effect of match nature, it was introduced as an adjustment variable, treated as a dummy variable, with a value of 0 for friendly matches and 1 for official matches. This variable was included as a fixed-effects factor and as a random-effects factor in the interaction. In all models, PL was considered the independent variable. Given the nature of linear mixed models, normality was assessed post hoc through the examination of residuals [28]. This assumption was verified using the Shapiro–Wilk and Kolmogorov–Smirnov tests.

To quantify the difference between groups for each time window, effect size (ES) was calculated using Cohen’s d. Descriptive statistics, linear mixed-effects models, and Bonferroni post hoc comparisons were performed using the SPSS software (Version 28, IBM Corp., Armonk, NY, USA), with statistical significance set at 95% (*p* < 0.05). Effect sizes and their corresponding 95% confidence intervals (CIs) were computed using a custom-configured Excel spreadsheet (Version 16.0, Microsoft Corp., Redmond, WA, USA). ES values were interpreted as trivial (≤0.20), small (0.21–0.60), moderate (0.61–1.20), large (1.21–2.00), very large (2.01–4.00), and extremely large (>4.00) [29] and were analyzed in conjunction with their 95% CIs to assess the precision and potential triviality of the estimates. If the 95% CI overlapped zero, the ES was interpreted as trivial, regardless of the point estimate.

Although the variable match nature was used as an adjustment variable to control for its effect, given that official matches for the elite team were unavailable, a second complementary analysis was conducted. In this second analysis, only friendly matches were considered: 8 matches from the elite team and 3 from the non-elite team. Similarly, player level was introduced as a fixed effect, the athlete as a random-effects factor, and age as a covariate or adjustment variable, following the steps previously described.

## 3. Results

**Figure 1:**
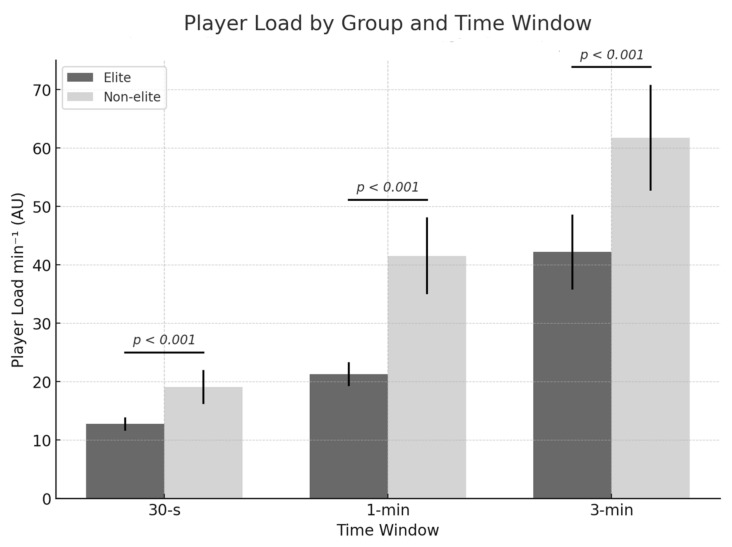
Player load values for each group across the time windows. The *p*-values for between-group differences are shown for each time window, derived from linear mixed models adjusted for age and match nature.

**Figure 2:**
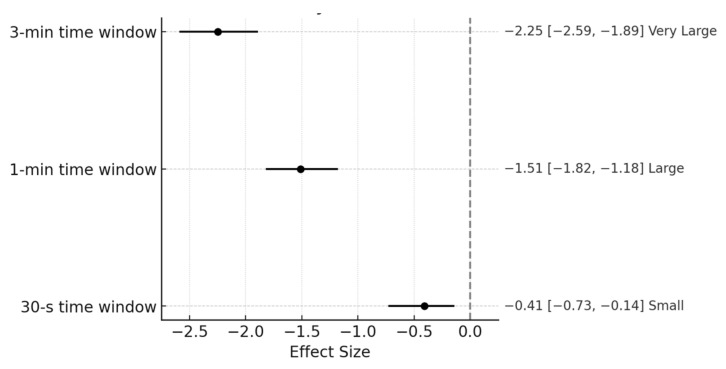
Effect size and CIs for each of the time windows.

The findings of the primary analysis are displayed through tables and figures. The mean, standard deviation, and coefficient of variation are shown for each group and measurement window in the PL variable (see Table 1 and Figure 1). Additionally, the differences found in the comparisons for each measurement window are presented, along with the effect size and CIs for each (See Table 2 and Figure 2).

Significant differences were found (*p* < 0.001) for all analyses conducted, with ES ranging from −0.41 to −2.11 (small—very large) and 95% CIs ranging from −0.70 to −0.12 (30-s), −1.78 to −1.16 (1-min), and −2.44 to −1.77 (3-min). All ESs were accompanied by 95% CIs, and none of them overlapped zero, indicating meaningful group differences.

The results of the secondary analysis are presented in the form of tables. For the PL variable, the mean, standard deviation, and coefficient of variation are reported for each group and measurement window (see Table 3). Furthermore, the results of the pairwise comparisons across the different measurement windows are displayed, including the effect sizes and corresponding CIs (see Table 4).

All analyses revealed statistically significant differences (*p* < 0.001), with effect sizes ranging from −2.68 to −5.06, indicating large to extremely large effects and 95% CIs ranging from −3.27 to −2.03 (30-s), −5.91 to −4.11 (1-min), and −4.03 to −2.64 (3-min).

## 4. Discussion

This study aimed to investigate the influence of player level (elite vs. non-elite) on the external PDs encountered by male basketball players over three distinct time windows (30-s, 1-min, and 3-min). Statistically significant differences were observed between the groups for the PL variable across all three time windows. Given the significant age difference between the elite (25.9 ± 3.2) and non-elite groups (20.72 ± 4.2; *p* < 0.05), age was included as a covariate to control for its potential effect on the findings. Furthermore, match nature was statistically adjusted as a dummy variable.

As previously noted, significant differences were found regarding the differences between groups. Across all measurement windows, the peak PL values attained by the non-elite athletes were higher than those of the elite players. In the longer windows, the differences were meaningfully greater, as indicated by the magnitude of the ES and 95% CIs not overlapping zero. The ES for the 30-s time window was qualitatively considered small (ES = −0.41; 95% CI = −0.70 to −0.12, *p* < 0.001). For the 1 min window, the ES was considered large (ES = −1.47; 95% CI = −1.78 to −1.16, *p* < 0.001). Finally, for the 3-min window, the ES obtained was the highest in qualitative terms, rated as very large (ES = −2.11; 95% CI = −2.44 to −1.77, *p* < 0.001). These findings have been related to those obtained in the study by Trapero et al. [21], in which it was observed that non-elite athletes performed a higher number of accelerations and decelerations compared to the elite group [21]. However, as mentioned in the introduction, that study did not use time windows but rather average values.

The only study we found that used time windows was the one comparing two elite teams, in this case, one from the Euroleague and the other from the Eurocup, with the latter being of a lower competitive level. In this study, despite both teams being elite-level, the Eurocup team achieved higher PDs in various peak and average physical performance variables compared to the Euroleague team [22], which aligns with the data obtained in the present study.

Regarding the other studies previously mentioned, which were analyzed using time–motion analysis, comparisons are somewhat more challenging due to the different measurement methodologies employed and the absence of time windows. In contrast to the findings of Scanlan et al. [18], where elite athletes exhibited higher external load values, our results showed lower PDs among elite players. However, although the comparison is neither precise nor reliable, it appears that the external load peaks, or “bursts”, as referred to by the authors, were higher in the sub-elite group [18], which seems to correlate with the higher PDs recorded by the mobile windows system used in the present study, which does not rely on average values in order to avoid underestimating external load. Finally, the results obtained by Sirotic et al. and Conte et al., in this case, with rugby players, could not be confirmed, as no significant differences were found between the elite and semi-elite groups in the variables analyzed [19,20].

Considering only the results from the second analysis, which involved friendly matches, the results follow the same trend. Across all three time windows, non-elite athletes reached significantly higher PL values (*p* < 0.001) compared to the elite group, with effect sizes greater than those observed in the main analysis. The values were considered very large for the 30-s (ES = −2.68; 95% CI = −3.27 to −2.03, *p* < 0.001) and 3-min (ES = −3.36; 95% CI = −4.03 to −2.64, *p* < 0.001) windows and extremely large for the 1-min window (ES = −5.06; 95% CI = −5.91 to −4.11, *p* < 0.001). These results may seem surprising, as it is often assumed that higher-level athletes will demonstrate superior physical performance. Firstly, it is important to consider that the complexity of a sport or activity does not depend solely on physical and/or coordinative factors [30]. Marteniuk’s model suggests that sports skill depends on perceptual ability, decision-making, and execution [30,31,32]. Therefore, physical factors would only address one part of these three critical elements. As mentioned in a previous study, higher-level competitive athletes did not exhibit higher PDs compared to those of lower levels or those with less significance within their team [33]. In relation to Marteniuk’s model, authors such as Sampaio et al. [34] have suggested that physical performance is influenced by cognitive factors. Higher-level athletes, in this case, NBA All-Star players, performed slower or less intense actions, but ones that were more effective, allowing them to better manage their physical resources by making better decisions during the game [34]. Decision-making and cognitive factors are fundamental to agility in team sports [35] and, more specifically, in basketball [36]. A study conducted with Australian state-level players concluded that decision-making time and response speed were critical determinants of athletes’ agility and that training methods aimed at improving decision-making should be integrated into training programs [37].

Elite male basketball players exhibit superior psychological skills, such as selective attention, memory retention, and environmental analysis, compared to lower-level athletes [38]. A study that conducted an electroencephalographic analysis comparing elite and non-elite basketball players concluded that cortical activity was greater in the elite group [39]. However, this study was performed under highly controlled conditions, analyzing only free-throw shooting, a closed skill. Thus, its findings cannot be directly extrapolated to our research context.

Various studies conducted on other sports also reveal cognitive differences between elite and non-elite athletes. Two trials involving young football players found that elite-level athletes exhibit better inhibitory control, greater cognitive flexibility, and enhanced metacognition, as well as superior self-regulation, which enables them to be more effective in their environment by being aware of their skills and weaknesses [40,41]. Finally, a study on hockey players indicated that the primary difference between elite and non-elite athletes was related to procedural knowledge, specifically in terms of decision-making and positioning [42].

Therefore, one possible hypothesis for the difference between elite and non-elite players in the present study is related to cognitive and decision-making factors. These factors can be developed and enhanced through tasks such as small-sided games—by modifying rules, playing spaces, or the number of players—or through more analytical drills, such as change-of-direction (COD) exercises involving responses to visual or auditory stimuli [43].

While physical and conditional factors are relevant, achieving higher PDs does not appear to be the sole determinant of high sports performance. In this study, the data show that ES is greater in longer windows. That is, as the measurement duration increases, the difference in PL becomes more pronounced. This finding aligns with previous observations. In shorter time windows, if a high or very high-intensity action is required, leading to increased demands, the difference between teams of different levels may not be as evident. However, this is not necessary throughout the entire game, as basketball is an intermittent sport [1,2]. Consequently, in periods of longer duration, greater differences may emerge, given that elite players might more effectively select the moments when such intensity should be applied, as opposed to other instances where it may not be necessary, thereby optimizing their movement efficiency [34]. Considering all the aforementioned aspects, it is crucial to acknowledge that basketball performance is multifactorial. It may depend on physical, technical, tactical, and contextual factors—such as the opponent’s characteristics or the game situation—as well as mental and psychological components [12]. Therefore, analyses that consider only limited factors should be interpreted with caution.

From an injury prevention standpoint, optimizing physical and cognitive efficiency could lower the cumulative mechanical load on musculoskeletal structures, thereby reducing the incidence of acute and overuse injuries [23,44]. Training approaches that combine high-intensity demands with decision-making tasks not only enhance performance but may also serve as a protective strategy for athlete longevity and well-being. Structured load management, individualized intensity monitoring, and task-specific perceptual–cognitive training should be emphasized as part of comprehensive athlete health programs.

From a health perspective, improving efficiency in high-intensity actions could help reduce cumulative fatigue and lower injury risk, especially in congested competition periods. Emphasizing cognitive–perceptual training alongside physical development may foster more intelligent effort distribution, preserving athletes’ well-being while sustaining high performance levels.

Two important limitations should be considered. First, the number of monitored games differed substantially between the groups: the non-elite team participated in 29 matches, while the elite team played only 8. Second, all games for the elite group were friendly matches, while the non-elite group played a mix of official and friendly matches. Although statistical adjustments were made for match nature, these differences could still contribute to the observed effects and should be taken into account when interpreting the results. Despite this, a previous study conducted on a non-elite sample found no significant differences in PL PDs between official and non-official matches [33]. However, there is limited scientific evidence analyzing this phenomenon from the perspective of MDS. Consequently, a second analysis was conducted exclusively with friendly matches from both teams to validate the proposed hypothesis. Another limitation of the present study is the mean age difference between the two samples. To address this, the possible effect of age on PL was considered by including it as an adjustment variable in the statistical analysis. Finally, an additional limitation is the lack of access to positional data (such as distances covered or running speeds), as well as the absence of analysis of other inertial parameters, including jumping actions. Such data would provide greater insight into the demands of external load and offer a more comprehensive comparison of the results.

These findings highlight the importance of tailoring training programs not merely to maximize physical output but to optimize it within the context of game situations. Coaches and strength and conditioning professionals should integrate high-intensity tasks with decision-making challenges—such as small-sided games or agility drills with perceptual stimuli—to better replicate the demands experienced during competition. Since elite players appear to regulate their efforts more efficiently across longer durations, promoting movement economy and tactical awareness may be more beneficial than simply increasing training volume or intensity.

From a future research perspective, it would be interesting to conduct this type of analysis by including other related variables, such as accelerations, decelerations, jumps, and running at different intensities. This could provide greater depth and richness to both the data and the understanding of this area of research. Furthermore, it would be advisable to monitor several different teams, both elite and non-elite, over more than one season, including both friendly and official matches.

## 5. Conclusions

The peak demands of PL experienced by non-elite players are significantly higher compared to elite players. While physical factors play a crucial role, they are not the most decisive in athletic performance. Higher-level athletes, in this case, elite players, appear to be more efficient or economical in executing high-intensity actions, making better decisions. This effect was more pronounced in longer windows. In the primary analysis, the 1-min window showed a large effect (ES = −1.47; 95% CI = −1.78 to −1.16, *p* < 0.001), and the 3-min window a very large effect (ES = −2.11; 95% CI = −2.44 to −1.77, *p* < 0.001), compared to the shortest window (30-s, ES = −0.41; 95% CI = −0.70 to −0.12, *p* < 0.001). In the secondary analysis (friendly matches only), effect sizes were even greater: ES = −5.06 for the 1-min window (95% CI = −5.91 to −4.11), ES = −3.36 for the 3-min window (95% CI = −4.03 to −2.64), and ES = −2.68 for the 30-s window (95% CI = −3.27 to −2.03), all with *p* < 0.001.

The primary practical application of this study lies in the value of understanding these data. Performance improvement does not stem solely from increasing the workload imposed on athletes. Training tasks should incorporate exercises that combine high intensity with decision-making and perceptual abilities in real-game situations. This approach enables athletes to optimize their movement efficiency, reducing physical exertion while achieving better results. Incorporating these strategies into athlete development programs may not only boost performance but also contribute to injury prevention and long-term health maintenance, ultimately supporting sustainable elite careers.

## Figures and Tables

**Table 1:** Descriptive statistics for each group and their corresponding time windows.

Descriptive											
30-s Time Window				1-min Time Window				3-min Time Window			
Variable	Group	Mean ± SD	%CV	Variable	Group	Mean ± SD	%CV	Variable	Group	Mean ± SD	%CV
Player Load (PL)	Elite	13.59 ± 1.15	9.2%	Player Load (PL)	Elite	22.38 ± 2.02	10.0%	Player Load (PL)	Elite	43.54 ± 6.41	15.7%
	Non-elite	19.39 ± 2.95	14.9%		Non-elite	42.71 ± 6.27	14.5%		Non-elite	62.80 ± 9.65	15.1%

Note: Age adjusted to 21.48 years. Match nature adjusted as dummy variable to 0.73 (0 = non-official; 1 = official).

**Table 2:** Comparison of the differences and effect size between groups for each of the time windows.

Mean Differences and Effect Sizes			
Sample Duration	Effect Size	95%CI	*p*
30-s time window,			
elite vs. non-elite	−0.41	−0.70, −0.12	<0.001
1-min time window,			
elite vs. non-elite	−1.47	−1.78, −1.16	<0.001
3-min time window,			
elite vs. non-elite	−2.11	−2.44, −1.77	<0.001

Note: Age adjusted to 21.48 years. Match nature adjusted as dummy variable to 0.73 (0 = non-official; 1 = official).

**Table 3:** Descriptive statistics for each group and their corresponding measurement windows in the non-official match sample.

Descriptive (Non-Official Matches)											
30-s Time Window				1-min Time Window				3-min Time Window			
Variable	Group	Mean ± SD	%CV	Variable	Group	Mean ± SD	%CV	Variable	Group	Mean ± SD	%CV
Player Load (PL)	Elite	12.73 ± 1.15	9.2%	Player Load (PL)	Elite	21.28 ± 2.02	10.0%	Player Load (PL)	Elite	42.18 ± 6.41	15.7%
	Non-elite	19.05 ± 2.93	14.9%		Non-elite	41.51 ± 6.58	15.1%		Non-elite	61.76 ± 9.06	15.1%

Note: Age adjusted to 24.09 years.

**Table 4:** Comparison of the differences and effect sizes between groups for each of the measurement windows in the friendly match sample.

Mean Differences and Effect Sizes (Non-Official Matches)			
Sample Duration	Effect Size	95%CI	*p*
30-s time window,			
elite vs. non-elite	−2.68	−3.27, −2.03	<0.001
1-min time window,			
elite vs. non-elite	−5.06	−5.91, −4.11	<0.001
3-min time window,			
elite vs. non-elite	−3.36	−4.03, −2.64	<0.001

Note: Age adjusted to 24.09 years.

## Data Availability

The original contributions presented in this study are included in the article; further inquiries can be directed to the authors.

## References

[1] Taylor J.B., Wright A.A., Dischiavi S.L., Townsend M.A., Marmon A.R. (2017). Activity Demands During Multi-Directional Team Sports: A Systematic Review. Sports Med..

[2] Sosa C., Lorenzo A., Trapero J., Ribas C., Alonso E., Jimenez S.L. (2021). Specific Absolute Velocity Thresholds during Male Basketball Games Using Local Positional System; Differences between Age Categories. Appl. Sci..

[3] Fox J.L., Scanlan A.T., Stanton R. (2017). A Review of Player Monitoring Approaches in Basketball: Current Trends and Future Directions. J. Strength Cond. Res..

[4] Alonso Pérez-Chao E., Portes R., Gómez M.Á., Parmar N., Lorenzo A., Jiménez-Sáiz S.L. (2023). A Narrative Review of the Most Demanding Scenarios in Basketball: Current Trends and Future Directions. J. Hum. Kinet..

[5] Halson S.L. (2014). Monitoring Training Load to Understand Fatigue in Athletes. Sports Med..

[6] Gonçalves G., Neta P., Ribeiro J., Guimaraes E. (2025). Internal and External Loads during Formal Training and Competition, Physical Capacities, and Technical Skills in Youth Basketball: A Comparison between Starters and Rotation Players. J. Hum. Kinet..

[7] Williams C., Rollo I. (2015). Carbohydrate Nutrition and Team Sport Performance. Sports Med..

[8] Calleja-González J., Terrados N., Mielgo-Ayuso J., Delextrat A., Jukic I., Vaquera A., Torres L., Schelling X., Stojanovic M., Ostojic S.M. (2016). Evidence-Based Post-Exercise Recovery Strategies in Basketball. Physician Sportsmed..

[9] Ochoa-Lácar J., Singh M., Bird S.P., Charest J., Huyghe T., Calleja-González J. (2022). How Sleep Affects Recovery and Performance in Basketball: A Systematic Review. Brain Sci..

[10] França C., Gomes B.B., Gouveia É.R., Ihle A., Coelho-E-silva M.J. (2021). The Jump Shot Performance in Youth Basketball: A Systematic Review. Int. J. Environ. Res. Public Health.

[11] Esteves P.T., Mikolajec K., Schelling X., Sampaio J. (2021). Basketball Performance Is Affected by the Schedule Congestion: NBA Back-to-Backs under the Microscope. Eur. J. Sport. Sci..

[12] Cao S., Geok S.K., Roslan S., Sun H., Lam S.K., Qian S. (2022). Mental Fatigue and Basketball Performance: A Systematic Review. Front. Psychol..

[13] Fox J.L., Conte D., Stanton R., McLean B., Scanlan A.T. (2021). The Application of Accelerometer-Derived Moving Averages to Quantify Peak Demands in Basketball: A Comparison of Sample Duration, Playing Role, and Session Type. J. Strength Cond. Res..

[14] Alonso E., Miranda N., Zhang S., Sosa C., Trapero J., Lorenzo J., Lorenzo A. (2020). Peak Match Demands in Young Basketball Players: Approach and Applications. Int. J. Environ. Res. Public Health.

[15] Cunningham D.J., Shearer D.A., Carter N., Drawer S., Pollard B., Bennett M., Eager R., Cook C.J., Farrell J., Russell M. (2018). Assessing Worst Case Scenarios in Movement Demands Derived from Global Positioning Systems during International Rugby Union Matches: Rolling Averages versus Fixed Length Epochs. PLoS ONE.

[16] McKay A.K.A., Stellingwerff T., Smith E.S., Martin D.T., Mujika I., Goosey-Tolfrey V.L., Sheppard J., Burke L.M. (2022). Defining Training and Performance Caliber: A Participant Classification Framework. Int. J. Sports Physiol. Perform..

[17] Swann C., Moran A., Piggott D. (2015). Defining Elite Athletes: Issues in the Study of Expert Performance in Sport Psychology. Psychol. Sport. Exerc..

[18] Scanlan A., Dascombe B., Reaburn P. (2011). A Comparison of the Activity Demands of Elite and Sub-Elite Australian Men’s Basketball Competition. J. Sports Sci..

[19] Sirotic A.C., Coutts A.J., Knowles H., Catterick C. (2009). A Comparison of Match Demands between Elite and Semi-Elite Rugby League Competition. J. Sports Sci..

[20] Conte D., Favero T.G., Niederhausen M., Capranica L., Tessitore A. (2017). Determinants of the Effectiveness of Fast Break Actions in Elite and Sub-Elite Italian Men’s Basketball Games. Biol. Sport..

[21] Trapero J., Trapero J., Sosa C., Zhang S., Portes R., Gómez-Ruano M.Á., Bonal J., Jiménez S.L., Lorenzo A. (2019). Comparison of the Movement Characteristics Based on Position-Specific Between Semi-Elite and Elite Basketball Players. Rev. de Psicol. Del Deporte.

[22] Ujaković F., Salazar H., Pleša J., Svilar L. (2024). Elite Basketball Game External Load Varies Between Different Teams and Competition. Kinesiology.

[23] Gabbett T.J. (2016). The Training-Injury Prevention Paradox: Should Athletes Be Training Smarter and Harder?. Br. J. Sports Med..

[24] Alonso Pérez-Chao E., Lorenzo A., Scanlan A., Lisboa P., Sosa C., Gómez M.Á. (2021). Higher Playing Times Accumulated Across Entire Games and Prior to Intense Passages Reduce the Peak Demands Reached by Elite, Junior, Male Basketball Players. Am. J. Mens. Health.

[25] Hodder R.W., Ball K.A., Serpiello F.R. (2020). Criterion Validity of Catapult ClearSky T6 Local Positioning System for Measuring Inter-Unit Distance. Sensors.

[26] Castellano J., Casamichana D., Calleja-González J., Román J.S., Ostojic S.M. (2011). Reliability and Accuracy of 10 Hz GPS Devices for Short-Distance Exercise. J. Sports Sci. Med..

[27] Bredt S.D.G.T., Chagas M.H., Peixoto G.H., Menzel H.J., Andrade A.G.P.D. (2020). Understanding Player Load: Meanings and Limitations. J. Hum. Kinet..

[28] Barr D.J., Levy R., Scheepers C., Tily H.J. (2013). Random Effects Structure for Confirmatory Hypothesis Testing: Keep It Maximal. J. Mem. Lang..

[29] Hopkins W.G., Marshall S.W., Batterham A.M., Hanin J. (2009). Progressive Statistics for Studies in Sports Medicine and Exercise Science. Med. Sci. Sports Exerc..

[30] Broadbent D.P., Causer J., Williams A.M., Ford P.R. (2015). Perceptual-Cognitive Skill Training and Its Transfer to Expert Performance in the Field: Future Research Directions. Eur. J. Sport. Sci..

[31] Marteniuk R.G. (1976). Cognitive Information Processes in Motor Short-Term Memory and Movement Production. Motor Control.

[32] Hayes K.C., Marteniuk R.G. (1976). Dimensions of Motor Task Complexity. Motor Control.

[33] Antoranz Y., Alonso-Pérez-Chao E., Tejero-González C.M., Salazar H., Del Campo-Vecino J., Jiménez-Sáiz S.L. (2024). Exploring External Peak Demands: The Influence of Contextual Factors on Male Basketball Players. Appl. Sci..

[34] Sampaio J., McGarry T., Calleja-González J., Jiménez Sáiz S., Schelling I Del Alcázar X., Balciunas M. (2015). Exploring Game Performance in the National Basketball Association Using Player Tracking Data. PLoS ONE.

[35] Paul D.J., Gabbett T.J., Nassis G.P. (2016). Agility in Team Sports: Testing, Training and Factors Affecting Performance. Sports Med..

[36] Morrison M., Martin D.T., Talpey S., Scanlan A.T., Delaney J., Halson S.L., Weakley J. (2022). A Systematic Review on Fitness Testing in Adult Male Basketball Players: Tests Adopted, Characteristics Reported and Recommendations for Practice. Sports Med..

[37] Scanlan A., Humphries B., Tucker P.S., Dalbo V. (2014). The Influence of Physical and Cognitive Factors on Reactive Agility Performance in Men Basketball Players. J. Sports Sci..

[38] Kioumourtzoglou E., Derri V., Tzetzis G., Theodorakis Y. (1998). Cognitive, Perceptual, and Motor Abilities in Skilled Basketball Performance. Percept. Mot. Ski..

[39] Keshvari F., Farsi A., Abdoli B. (2024). Investigating the EEG Profile of Elite and Non-Elite Players in the Basketball Free Throw Task. J. Mot. Behav..

[40] Huijgen B.C.H., Leemhuis S., Kok N.M., Verburgh L., Oosterlaan J., Elferink-Gemser M.T., Visscher C. (2015). Cognitive Functions in Elite and Sub-Elite Youth Soccer Players Aged 13 to 17 Years. PLoS ONE.

[41] Toering T.T., Elferink-Gemser M.T., Jordet G., Visscher C. (2009). Self-Regulation and Performance Level of Elite and Non-Elite Youth Soccer Players. J. Sports Sci..

[42] Elferink-Gemser M.T., Kannekens R., Lyons J., Tromp Y., Visscher C. (2010). Knowing What to Do and Doing It: Differences in Self-Assessed Tactical Skills of Regional, Sub-Elite, and Elite Youth Field Hockey Players. J. Sports Sci..

[43] Serpell B.G., Young W.B., Ford M. (2011). Are the Perceptual and Decision-Making Components of Agility Trainable? A Preliminary Investigation. J. Strength Cond. Res..

[44] Soligard T., Schwellnus M., Alonso J.M., Bahr R., Clarsen B., Dijkstra H.P., Gabbett T., Gleeson M., Hägglund M., Hutchinson M.R. (2016). How Much Is Too Much? (Part 1) International Olympic Committee Consensus Statement on Load in Sport and Risk of Injury. Br. J. Sports Med..

